# Spleen tyrosine kinase inhibition is an effective treatment for established vasculitis in a pre-clinical model

**DOI:** 10.1016/j.kint.2019.12.014

**Published:** 2020-06

**Authors:** Stephen P. McAdoo, Maria Prendecki, Anisha Tanna, Tejal Bhatt, Gurjeet Bhangal, John McDaid, Esteban S. Masuda, H. Terence Cook, Frederick W.K. Tam, Charles D. Pusey

**Affiliations:** 1 Centre for Inflammatory Disease, Department of Medicine, Imperial College London, London UK; 2 Rigel Pharmaceuticals, South San Francisco, California, USA

**Keywords:** ANCA, experimental models, glomerulonephritis, kinase inhibitors, SYK, vasculitis

## Abstract

The anti-neutrophil cytoplasm antibody (ANCA)-associated vasculitides (AAV) are a group of life-threatening multi-system diseases characterized by necrotising inflammation of small blood vessels and crescentic glomerulonephritis. ANCA are thought to play a direct pathogenic role. Previous studies have shown that spleen tyrosine kinase (SYK) is phosphorylated during ANCA-induced neutrophil activation *in vitro*. However, the role of SYK *in vivo* is unknown. Here, we studied its role in the pathogenesis of experimental autoimmune vasculitis, a pre-clinical model of myeloperoxidase-ANCA-induced pauci-immune systemic vasculitis in the Wistar Kyoto rat. Up-regulation of SYK expression in inflamed renal and pulmonary tissue during early autoimmune vasculitis was confirmed by immunohistochemical and transcript analysis. R406, the active metabolite of fostamatinib, a small molecule kinase inhibitor with high selectivity for SYK, inhibited ANCA-induced pro-inflammatory responses in rat leucocytes *in vitro*. In an *in vivo* study, treatment with fostamatinib for 14 days after disease onset resulted in rapid resolution of urinary abnormalities, significantly improved renal and pulmonary pathology, and preserved renal function. Short-term exposure to fostamatinib did not significantly affect circulating myeloperoxidase-ANCA levels, suggesting inhibition of ANCA-induced inflammatory mechanisms *in vivo*. Finally, SYK expression was demonstrated within inflammatory glomerular lesions in ANCA-associated glomerulonephritis in patients, particularly within CD68^+^ve monocytes/macrophages. Thus, our data indicate that SYK inhibition warrants clinical investigation in the treatment of AAV.

## Graphical abstract

**Figure:**
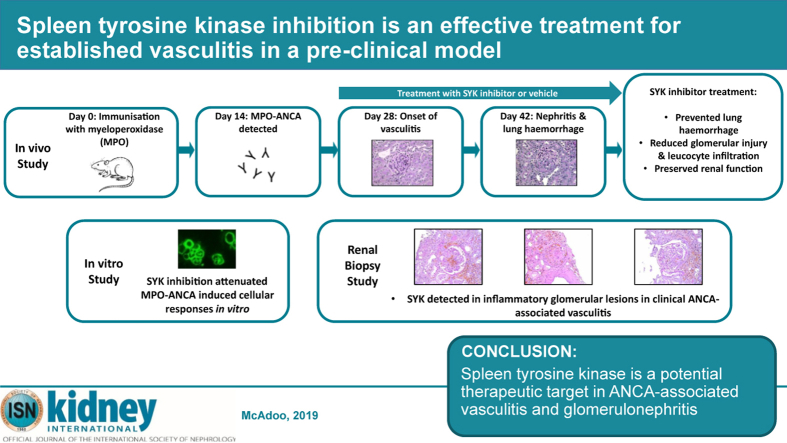

**Translational Statement**

The anti-neutrophil cytoplasm antibody (ANCA)–associated vasculitides are a group of rare diseases characterized by the inflammation of blood vessels, crescentic glomerulonephritis, and lung hemorrhage. Current treatments are not completely effective, as many patients develop refractory or relapsing disease, or treatment-related toxicities. This article investigates the role of spleen tyrosine kinase (SYK), an immunoreceptor-associated signaling protein, in the pathogenesis of ANCA vasculitis and its potential as a therapeutic target. In a preclinical model, SYK upregulation was identified at sites of renal and pulmonary inflammation, and treatment with an SYK inhibitor was completely effective in treating established lung and kidney disease, without hematological toxicity. SYK expression is also confirmed in glomerular inflammatory lesions in human ANCA-associated vasculitides. Clinical studies of SYK inhibition in IgA nephropathy are ongoing, and these novel data suggest that this approach should also be considered in ANCA vasculitis.

Spleen tyrosine kinase (SYK) is a cytosolic protein tyrosine kinase that is expressed in most leucocyte populations, where it has diverse immune functions. It has a well characterized role in mediating signaling from classical immunoreceptors such as the B-cell receptor and the Fc receptor, and also from some integrins and C-type lectins.^1^ Targeting SYK has thus emerged as a potential treatment approach for a variety of immune and inflammatory diseases,^2^ and a number of specific SYK inhibitors are in development.^3^ We have previously shown that fostamatinib, a small molecule kinase inhibitor with selectivity for SYK, is an effective treatment in experimental models of immune-complex glomerulonephritis.^4^^,^^5^ SYK also mediates proinflammatory responses induced by IgA1 derived from patients with IgA nephropathy,^6^ and full results from a Phase II clinical study assessing SYK inhibition with fostamatinib in proliferative IgA nephropathy are awaited (NCT02112838).

The anti-neutrophil cytoplasm antibody (ANCA)–associated vasculitides (AAV) are a group of life-threatening multi-system diseases characterized by necrotizing inflammation of small blood vessels and pauci-immune crescentic glomerulonephritis, in which ANCA are thought to play a directly pathogenic role.^7^ It has previously been shown that SYK activation occurs following ANCA-induced neutrophil activation,^8^ suggesting that SYK inhibition may be a potential therapeutic approach in AAV, though *in vivo* data are lacking. Here, we have investigated the effect of SYK inhibition in an experimental model of myeloperoxidase (MPO)-ANCA–induced systemic vasculitis (experimental autoimmune vasculitis [EAV]) that was developed in our laboratory.^9^^,^^10^ It is characterized by ANCA-induced enhancement of leucocyte–endothelial cell interactions and the development of both alveolar hemorrhage and necrotizing glomerulonephritis by 4 weeks after disease induction. In contrast to our previous studies in immune-complex glomerulonephritis, this model has a distinct pauci-immune mechanism of tissue injury, similar to that in AAV.

## Results

### SYK is expressed and activated at sites of disease in experimental autoimmune vasculitis

**Figure 1:**
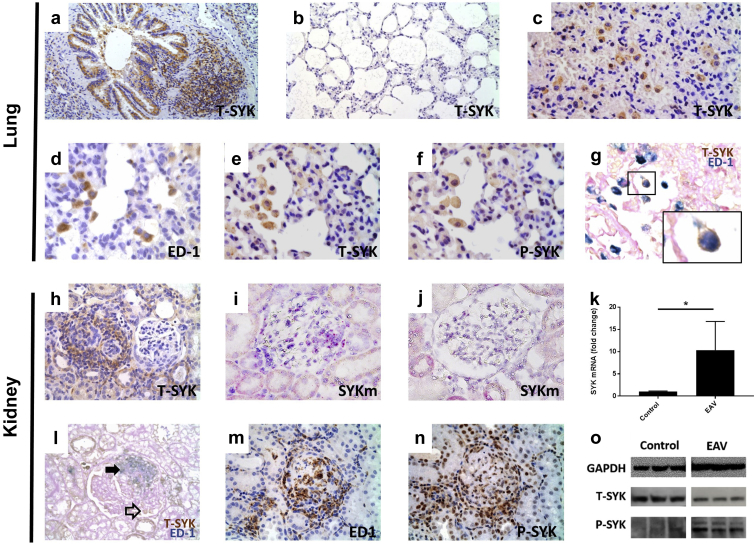
**Spleen tyrosine kinase (SYK) is expressed and activated at sites of disease in experimental autoimmune vasculitis.** Immunohistochemical staining for total (T)-SYK, phosphorylated (P)-SYK, and ED-1 (rat homologue of CD68) in healthy and diseased rat lung and renal tissue 6 weeks after induction of experimental autoimmune vasculitis (EAV). (**a,b**) T-SYK detection in a healthy lung, demonstrating (**a**) SYK expression in large airway cuboidal epithelial cells and associated lymphoid tissue, but (**b**) minimal SYK detection in alveolar squamous epithelium. (**c**) T-SYK detection in inflamed lung tissue, demonstrating a population of large mononuclear cells that are positive for SYK, with alveolar consolidation by erythrocytes. (**d–g**) Staining of serial sections of lung tissue showing an alveolar lumen containing mononuclear cells positive for (**d**) ED-1, (**e**) T-SYK, and (**f**) P-SYK. Double staining confirms co-localization of T-SYK (brown) and ED-1 (blue) in these alveolar cells. (**h**) Glomerular T-SYK detection in adjacent crescentic and normal glomeruli in nephritic kidney tissue, demonstrating SYK detection within proliferative lesions in diseased glomeruli, although no expression in preserved, non-inflamed glomeruli. (**i,j**) RNAScope (Advanced Cell Diagnostics, Newark, CA) *in situ* hybridization for SYK mRNA, stained in purple, in (**i**) nephriritic and (**j**) normal glomeruli. (**k**) SYK mRNA expression in rat renal tissue 6 weeks after induction of EAV and in control rats immunized with complete Freunds adjuvant alone, confirming upregulation of SYK expression in nephritic tissue (n = 4 per group; statistical comparison by Mann-Whitney test; ∗*P* < 0.05). (**l**) Double staining demonstrating co-localization of ED-1 (blue) and T-SYK (brown) with a segmental area of inflammation in a nephritic glomerulus (solid arrowhead), and a small population of T-SYK+ ED-1 cells (open arrowhead). (**m**) ED-1 and (**n**) P-SYK staining in sequential sections from nephritic rat tissue, suggesting co-localization of SYK activation with infiltrating ED-1 expressing monocytes/macrophages. (**o**) Immunoblotting for T-SYK and P-SYK in whole kidney tissue, confirming upregulation of SYK phosphorylation during EAV compared to complete Freunds adjuvant control animals. (Representative photomicrographs, original magnification [**a**] ×100, [**b**] ×200, and [**c–o**] ×400, all performed with hematoxylin counterstain, except double-stain sections, which were counterstained with periodic acid–Schiff alone, without hematoxylin, to allow clear visualization of blue immunohistochemical stain). GAPDH, glyceraldehyde-3-phosphate dehydrogenase. To optimize viewing of this image, please see the online version of this article at www.kidney-international.org.

We performed immunohistochemical staining for total (T)- and activated (i.e., phosphorylated [P]-) SYK. In healthy rat lung tissue, this analysis demonstrated that T-SYK was expressed in large airway cuboidal epithelial cells and associated lymphoid tissue (Figure 1a), consistent with previously described patterns of SYK expression in hematopoetic and some epithelial cell types.^11^ There was minimal T-SYK detection in alveolar squamous epithelium (Figure 1b). In lung tissue taken from animals 6 weeks after induction of EAV (Figure 1c), alveolar lumens were consolidated with erythrocytes, consistent with the development of lung hemorrhage. In addition, large mononuclear cells with cytoplasmic T-SYK expression were seen. Staining of serial sections identified a population of mononuclear cells positive for ED-1 (the rat homologue of CD68), T-SYK, and P-SYK (Figure 1d–f, respectively) in diseased lung, and dual staining confirmed T-SYK expression in ED-1+ve cells (Figure 1g), suggesting an infiltrating population of monocytes/macrophages expressing activated SYK at sites of alveolar hemorrhage. A small number of T-SYK+ve ED-1-ve cells were also observed, suggesting additional cell populations that express SYK in this model, potentially lymphocytes or neutrophils. As previously described, in normal rat kidney tissue, T-SYK was detected in distal tubular epithelial cells but not in normal glomeruli. In kidney tissue taken from animals with established EAV, T-SYK was detected within inflamed glomeruli, particularly within areas of endocapillary proliferation and crescent formation, whereas there was no SYK detection in unaffected glomeruli (Figure 1h). Upregulation of SYK expression was confirmed by the finding of increased SYK mRNA in diseased renal tissue, by both *in situ* hybridization (Figure 1i and j) and by real-time quantitative polymerase chain reaction (RT-qPCR; Figure 1k). Dual staining showed co-localization of T-SYK and ED-1+ve cells within inflammatory glomerular lesions (Figure 1l). As observed in lung tissue, a small population of T-SYK+ve ED-1–ve cells was seen in some glomeruli. Staining of serial sections suggested that P-SYK localizes to infiltrating ED-1+ve monocytes/macrophages in and around glomeruli (Figure 1m and n). P-SYK staining in kidney sections was both cytoplasmic and nuclear; SYK is known to have a nuclear localization signal in B lymphocytes,^12^ and we have previously described nuclear staining for P-SYK in human kidney disease.^13^ In order to confirm SYK phosphorylation in EAV kidney tissue, we performed immunoblotting for P-SYK in kidney cortex, and showed upregulation compared with control kidney tissue (Figure 1o).

### SYK inhibition reduces the severity of lung hemorrhage in experimental autoimmune vasculitis

These immunohistochemical data suggest that SYK is expressed and activated at sites of renal and pulmonary inflammation in EAV. We therefore sought to examine the effect of SYK inhibition using fostamatinib *in vivo*. EAV was induced in Wistar Kyoto rats (n = 8 per group) by immunization with recombinant human MPO in complete Freunds adjuvant (CFA). After onset of vasculitis was confirmed by the development of proteinuria and hematuria 4 weeks after disease induction, animals were treated with fostamatinib of 30 mg/kg, 20 mg/kg, or vehicle preparation by twice daily oral gavage, and assessed for disease severity at 6 weeks, after a total period of 14 days of treatment with drug or vehicle. Control animals were immunized with CFA alone and followed until 6 weeks (n = 4). To obtain comparative lung and renal histology at treatment initiation (week 4), an additional group of animals was used after induction of EAV (n = 6).

**Figure 2:**
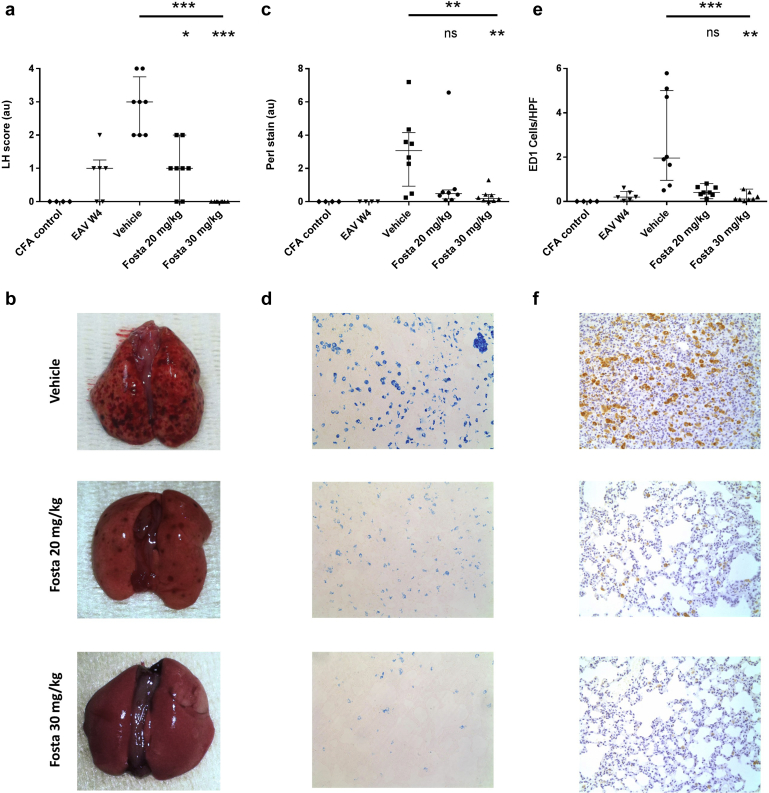
**Spleen tyrosine kinase (SYK) inhibition reduces the severity of lung hemorrhage in experimental autoimmune vasculitis (EAV).** (**a**) Lung hemorrhage severity scores in control, vehicle, and fostamatinib (Fosta)-treated rats, assessed at treatment initiation (week 4 [W4]) and at cull 6 weeks after disease induction, with lower panel (**b**) showing representative macroscopic lung pathology. (**c**) Quantification for hemosiderin deposition in lung tissue at treatment initiation (W4) and 6 weeks after disease induction, with representative photomicrographs (**d**) demonstrating staining for Perls’ Prussian Blue (without counterstain; original magnification ×200). (**e**) Quantification for ED-1 (rat homologue of CD68)–positive cells in lung tissue at treatment initiation (W4) and 6 weeks after disease induction, with (**f**) representative photomicrographs of ED-1 staining in lung tissue (with hematoxylin counterstain; original magnification ×200). All data are reported as median ± interquartile range, statistical comparison by Kruskal-Wallis test, with Dunn’s post-test comparison to vehicle group (lower indicator); ∗*P* < 0.05; ∗∗*P* < 0.01; ∗∗∗*P* < 0.001. CFA, complete Freunds adjuvant; LH, lung hemorrhage; ns, not significant. To optimize viewing of this image, please see the online version of this article at www.kidney-international.org.

Four weeks after induction of EAV, the majority of animals had macroscopic evidence of lung hemorrhage, and in vehicle-treated animals, this progressed to severe lung hemorrhage in all animals by week 6 (Figure 2a). Fostamatinib treatment resulted in a dose-dependent reduction in the severity of pulmonary hemorrhage, as determined by macroscopic assessment of lung tissue at the time of cull (Figure 2a and b; median lung hemorrhage score 3, 1, and O for vehicle, 20 mg/kg, and 30 mg/kg, respectively, *P* < 0.0001), and by histologic assessment for hemosiderin-laden cells in lung parenchyma (Figure 2c and d; median Perls’ stain detection 3.1, 0.5, and 0.2 au for vehicle, 20 mg/kg, and 30 mg/kg, respectively, *P* = 0.009). In addition, there was a dose-dependent reduction in ED-1 positive macrophage infiltration (Figure 2e and f; median 2.0, 0.4, and 0.1 ED-1 cells/field for vehicle, 20 mg/kg, and 30 mg/kg, respectively, *P* < 0.0001). Notably, treatment with fostamatinib 30 mg/kg led to complete reversal of lung hemorrhage in all rats.

### SYK inhibition reduces the severity of renal damage in experimental autoimmune vasculitis

**Figure 3:**
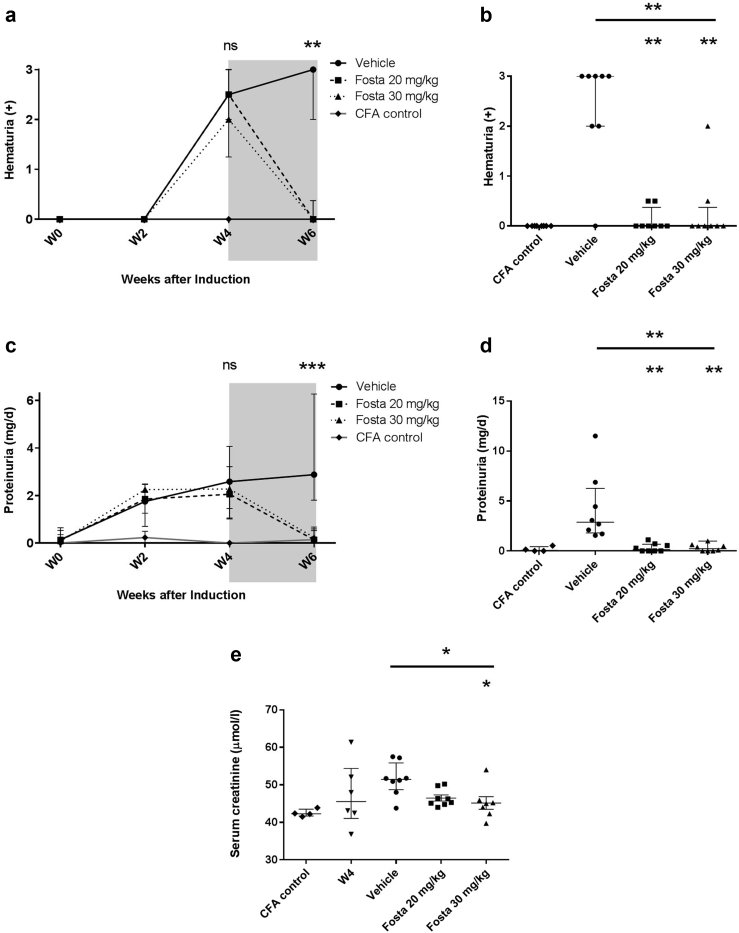
**Spleen tyrosine kinase inhibition reduces urinary abnormalities and improves renal function in experimental autoimmune vasculitis.** (**a**) Hematuria and (**c**) proteinuria, from day-of-disease induction (W0) to week 6 (W6), with the fostamatinib (Fosta) treatment period shaded in gray, showing complete resolution of urinary abnormalities at both treatment doses, and persistent hematuria and proteinuria in vehicle-treated rats, at W6. (**b,d**) Individual results at W6 for hematuria and proteinuria, respectively. (**e**) Serum creatinine measurements at treatment initiation (W4) and 6 weeks after disease induction, showing dose-dependent preservation of excretory renal function with fostamatinib treatment. All data are reported as median ± interquartile range, statistical comparison by Kruskal-Wallis test, with Dunn’s post-test comparison to vehicle group (lower indicator); ∗*P* < 0.05; ∗∗*P* < 0.01; ∗∗∗*P* < 0.001. ns, not significant.

**Figure 4:**
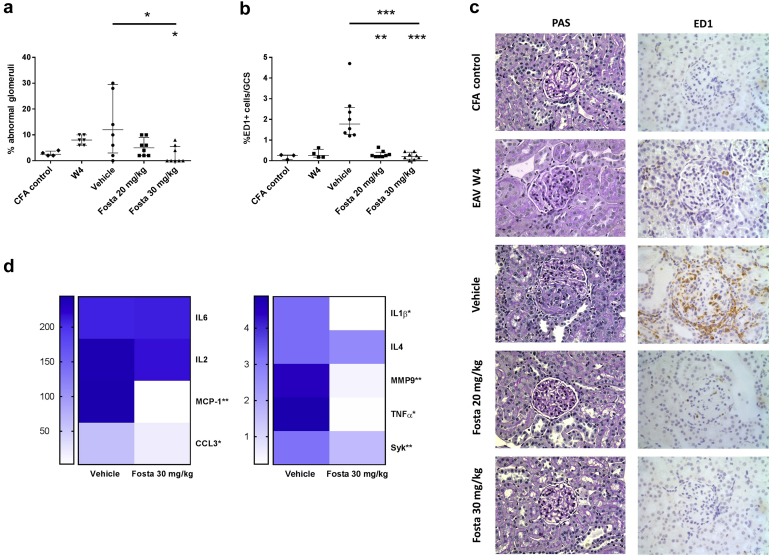
**Spleen tyrosine kinase (SYK) inhibition improves renal histopathology in experimental autoimmune vasculitis (EAV).** (**a**) Quantification of glomerular abnormalities at treatment initiation (week 4 [W4]) and at 6 weeks after disease induction, with (**c**) representative photomicrographs demonstrating focal necrotizing glomerulonephritis and crescent formation in vehicle-treated animals and preserved glomerular histology after fostamatinib (Fosta) treatment (periodic acid–Schiff [PAS]–stained sections, original magnification ×400). (**b**) Quantification of ED-1 (rat homologue of CD68)–positive cells infiltrating glomeruli at treatment initiation (W4) and at 6 weeks after disease induction, showing a dose-dependent reduction with fostamatinib treatment, with representative photomicrographs demonstrating immunoperoxidase staining for ED-1 (**b**; with hematoxylin counterstain; original magnification ×400). (**d**) Heat-map indicating changes in intra-renal gene expression following treatment with fostamatinib, as determined by real-time quantitative polymerase chain reaction and expressed as fold-change compared to normal kidney tissue. All data are reported as median ± interquartile range; statistical comparison by Mann-Whitney or Kruskal-Wallis test, with Dunn’s post-test comparison to vehicle group (lower indicator); ∗*P* < 0.05; ∗∗*P* < 0.01; ∗∗∗*P* < 0.0001. CCL3, C–C motif chemokine ligand 3; CFA, complete Freunds adjuvant; MCP-1, monocyte chemoattractant protein 1; MMP9, matrix metallopeptidase 9; ns, not significant; TNF, tumor necrosis factor. To optimize viewing of this image, please see the online version of this article at www.kidney-international.org.

Introduction of fostamatinib treatment 4 weeks after disease induction was associated with rapid resolution of urinary abnormalities during the 2-week treatment period. Compared to vehicle-treated animals, there were significant reductions in both hematuria (Figure 3a and b; median dipstick hematuria 3, 0, and 0 for vehicle, 20 mg/kg, and 30 mg/kg, respectively, *P* = 0.002) and proteinuria (Figure 3c and d; median proteinuria 2.8, 0.7, and 0.2 mg/day for vehicle, 20 mg/kg, and 30 mg/kg, respectively, *P* = 0.0004). These improvements were associated with a dose-dependent preservation of excretory renal function (Figure 3e; median serum creatinine 51.5, 45.8, and 45 μmol/l for vehicle, 20 mg/kg, and 30 mg/kg, respectively, *P* = 0.027). Histologic examination of renal tissue at 4 weeks, prior to treatment initiation, confirmed features of early proliferative glomerulonephritis and macrophage infiltration, that by 6 weeks progressed to focal necrotizing and crescentic glomerulonephritis in vehicle-treated animals (Figure 4). There was a dose-dependent reduction in proliferative glomerular lesions (Figure 4a and c; median 12%, 5%, and 0% glomerular abnormalities for vehicle, 20 mg/kg, and 30 mg/kg, respectively, *P* = 0.026) and ED-1+ve monocyte/macrophage glomerular infiltration (Figure 4b and c; median 1.8, 0.3, and 0.2 ED-1+ cells per glomerular cross-section for vehicle, 20 mg/kg, and 30 mg/kg, respectively, *P* = 0.0004). In keeping with reduced glomerular cell number, analysis of intra-renal gene expression (Figure 4d) confirmed a significant reduction in monocyte/macrophage-associated proinflammatory cytokine and enzyme production (monocyte chemoattractant protein–1 [MCP-1], C–C motif chemokine ligand 3 [CCL3], Il-1β, tumor necrosis factor– α [TNFα], matrix metallopeptidase 9 [MMP9]) with fostamatinib treatment, without a significant effect on the expression of T cell–associated cytokines (IL-2, IL-6, IL-4). There was a corresponding decrease in intra-renal SYK expression following fostamatinib treatment.

### Fostamatinib treatment does not affect circulating MPO-ANCA levels in experimental autoimmune vasculitis, but it may inhibit MPO-ANCA–induced cellular responses

**Figure 5:**
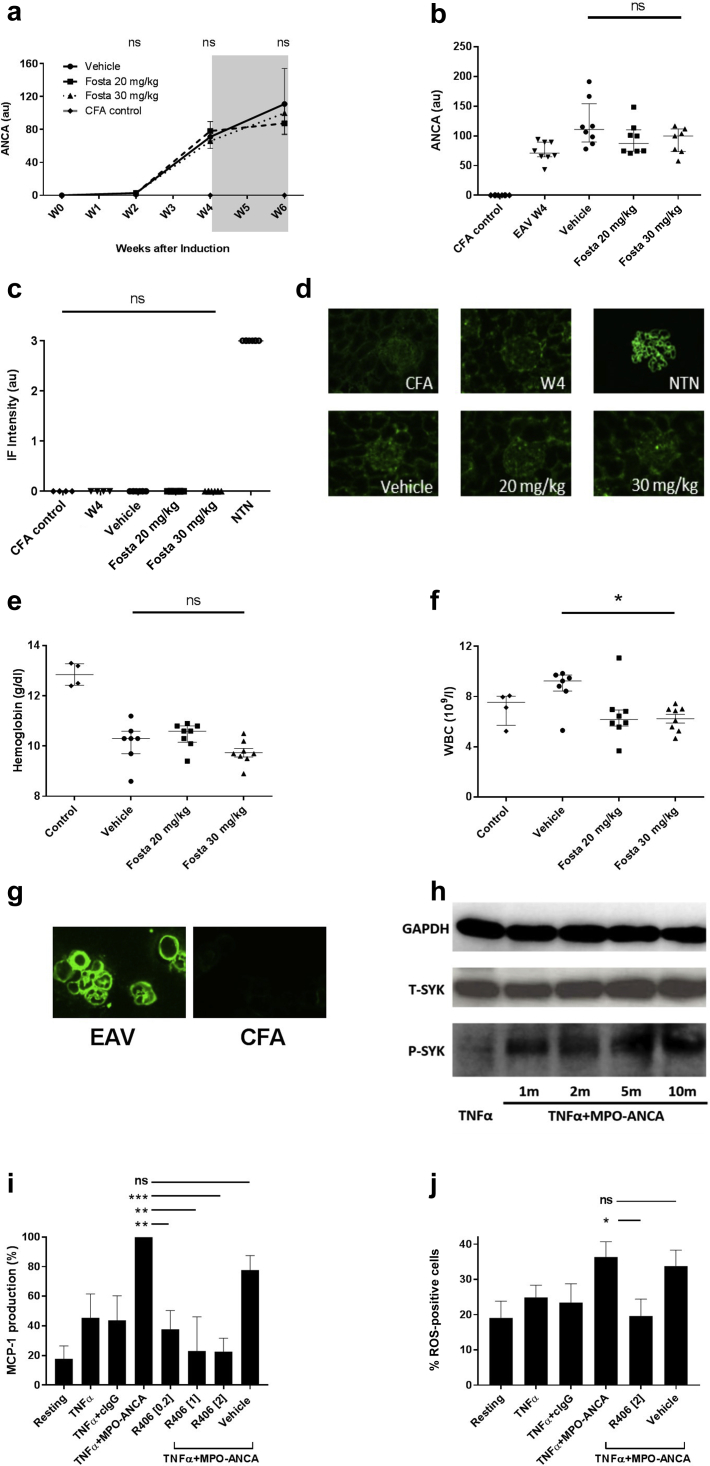
**Short-term fostamatinib (Fosta) treatment has minimal effects on myeloperoxidase–anti-neutrophil cytoplasm antibody(MPO-ANCA) production in experimental autoimmune vasculitis (EAV), although it inhibits MPO-ANCA–induced cellular responses *in vitro*.** (**a**) MPO-ANCA titres from day of disease induction (week 0 [W0]) to week 6 (W6), with the fostamatinib treatment period shaded in gray, showing no significant effect of treatment on circulating autoantibody levels. (**b**) Individual MPO-ANCA levels at W4 and W6. (**c**) Quantification of immunofluorescence (IF) staining for deposited IgG in glomeruli at treatment initiation (W4) and at 6 weeks after disease induction, confirming no difference in deposited IgG before or after fostamatinib treatment, with (**d**) representative immunofluorescence staining. Positive control tissue from animals after induction of nephrotoxic nephritis (NTN) included for comparison (all images, original magnification ×400). (**e,f**) Hematological indices following fostamatinib treatment in EAV, including (**e**) hemoglobin concentration and (**f**) white blood cell (WBC) count in each treatment group 6 weeks after disease induction. Fostamatinib treatment did not significantly alter hemoglobin concentrations. WBC counts were modestly reduced in fostamatinib-treated animals compared to vehicle-treated controls, although not beyond those of complete Freunds adjuvant (CFA)–alone immunized animals. (**g**) Indirect immunofluorescence on ethanol-fixed undifferentiated rat bone marrow cells using EAV and CFA control serum, confirming reactivity of EAV serum for both mononuclear and polymorphonuclear cells. (**h**) Immunoblotting confirms upregulation of SYK phosphorylation in undifferentiated bone marrow cells following priming with tumor necrosis factor α (TNFα) and stimulation with MPO-ANCA IgG. (**i,j**) Cellular responses by undifferentiated rat bone marrow cells following stimulation with TNFα, MPO-ANCA IgG, and control IgG (cIgG), alone or in combination, and following pretreatment with the active metabolite of fostamatinib, R406, or vehicle, showing (**i**) dose-dependent reduction in monocyte chemoattractant protein–1 (MCP-1) production (standardized results expressed as mean of 4 replicate experiments), and (**j**) inhibition of reactive oxygen species (ROS) generation, following R406 treatment. R406 exposure did not affect cell survival (Supplementary Figure S2). All data are reported as median ± interquartile range, statistical comparison by Kruskal-Wallis test, with Dunn’s post-test comparison to vehicle group (lower indicator); ∗*P* < 0.05; ∗∗*P* < 0.01; ∗∗∗*P* < 0.0001. GAPDH, glyceraldehyde-3-phosphate dehydrogenase; ns, not significant; SYK, spleen tyrosine kinase. To optimize viewing of this image, please see the online version of this article at www.kidney-international.org.

Short-term fostamatinib treatment did not significantly affect circulating MPO-ANCA levels in this study (Figure 5a and b; median MPO-ANCA titre 6 weeks after disease induction—111, 87, and 99 au for vehicle, 20 mg/kg, and 30 mg/kg, respectively, *P* = 0.19). Nor was there a significant effect on IgG subclass following treatment (Supplementary Figure S1). Direct immunofluorescence for deposited IgG in renal tissue demonstrated a pauci-immune pattern of renal injury and confirmed no difference in glomerular immune deposits between control animals and any treatment group (Figure 5c and d).

Fostamatinib treatment did not significantly depress hemoglobin concentrations compared to vehicle-treated animals (Figure 5e). However, there was a mild reduction in peripheral blood white cell count following fostamatinib treatment compared to vehicle-treated animals, consistent with previous reports that have attributed this phenomenon to a margination effect (Figure 5f; median white blood cell count 6 weeks after disease induction—9.2, 6.2, and 6.5 x 10^9^/l for vehicle, 20 mg/kg, and 30 mg/kg, respectively, *P* = 0.047). However, white blood cell counts were not reduced beyond those of control animals immunized with CFA alone (Figure 5f).

Although there was no difference in circulating MPO-ANCA levels after treatment, we observed that SYK inhibition could inhibit MPO-ANCA–induced cellular responses. Myeloid progenitors express high levels of the ANCA antigen MPO,^14^ and it has been shown that primed rat bone marrow–derived neutrophils and monocytes produce proinflammatory cytokines following ANCA stimulation.^15^ We confirmed that EAV serum is reactive to bone marrow mononuclear and polymorphonuclear cells by indirect immunofluorescence (Figure 5g), and that SYK is phosphorylated following stimulation of bone-marrow cells with rat MPO-ANCA (Figure 5h). We then sought to examine effects of SYK inhibition using R406, the active metabolite of fostamatinib, on MPO-ANCA–induced responses *in vitro*. After TNFα-priming, undifferentiated bone marrow cells produced high levels of MCP-1 following stimulation with rat-derived ANCA-IgG (whereas control IgG had no additional effect). Pretreatment with R406 was associated with a dose-dependent reduction in MCP-1 production (Figure 5i; 62%, 77%, and 75% reduction with 0.2, 1.0, and 2.0 μM R406, respectively, *P* = 0.0004). There was a similar reduction in production of reactive oxygen species, induced by MPO-ANCA following pretreatment with R406 (Figure 5j).

### SYK is expressed in inflammatory lesions in human ANCA-associated glomerulonephritis (AAGN)

**Figure 6:**
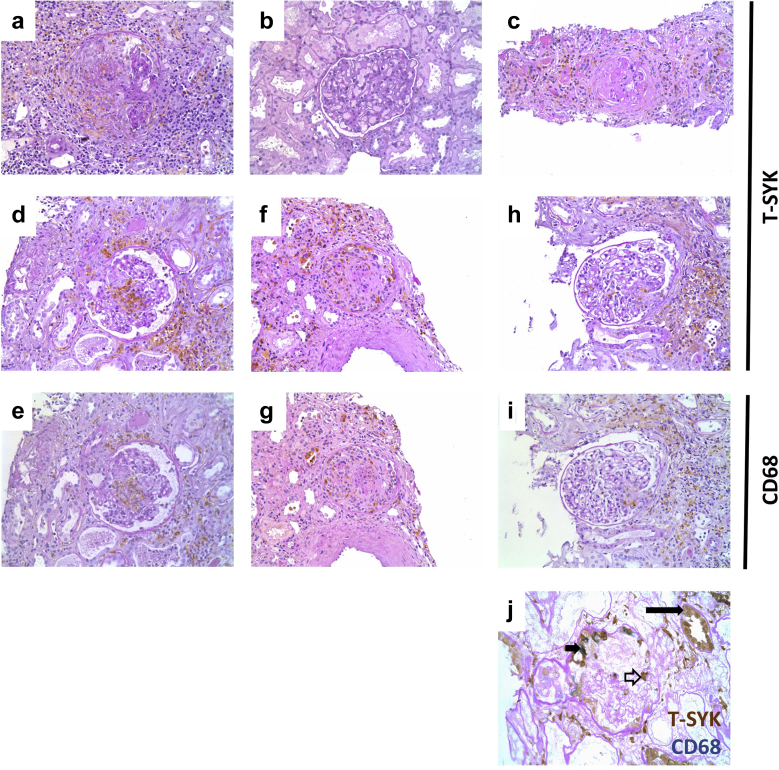
**Spleen tyrosine kinase (SYK) is expressed in inflammatory lesions in human anti-neutrophil cytoplasm antibody (ANCA)–associated glomerulonephritis.** Immunohistochemical staining for total (T)-SYK in ANCA-associated glomerulonephritis (AAGN) demonstrates SYK expression within inflammatory glomerular lesions including (**a**) crescents, but not within (**b**) normal or (**c**) sclerotic glomeruli. Staining of paired sections (**d–i**) for CD68 and T-SYK indicates significant co-localization of T-SYK to CD68-positive macrophages (**d,e**) within areas of glomerular fibrinoid necrosis, (**f,g**) extra-capillary proliferation, and (**h,i**) periglomerular inflammation. (**j**) Double staining for T-SYK (brown) and CD68 (blue) indicates distal tubular epithelial cell staining for T-SYK, as previously described (long solid arrowhead). Within glomeruli, there was significant co-localization of T-SYK and CD68 (short solid arrowhead) in cells within crescents. In addition, occasional T-SYK+ CD68– cells were observed (short open arrowhead). Photomicrographs, original magnification ×200 (**a–i**) and ×400 (**j**), all performed with hematoxylin and periodic acid–Schiff counterstain, except (**j**), which was counterstained with periodic acid–Schiff alone, without hematoxylin, to allow clear visualization of the blue immunohistochemical stain. To optimize viewing of this image, please see the online version of this article at www.kidney-international.org.

We and others have demonstrated that SYK expression is upregulated, and that SYK is activated, at sites of glomerular inflammation in renal AAV.^13^^,^^16^ Our previous studies, however, were conducted using a commercially available rabbit polyclonal antibody that is no longer available. We therefore have validated our previous description of SYK expression in AAGN using an alternative mouse mouse monoclonal antibody directed against human SYK (Figure 6). SYK was detected within inflammatory lesions in AAGN, including areas of glomerular crescent formation (Figure 6a and f), fibrinoid necrosis (Figure 6d), and periglomerular inflammation (H), but not within normal or sclerotic glomeruli (Figure 6b and c), suggesting that it is a feature of active disease. Staining of serial sections indicates significant co-localization of SYK to CD68+ve cells in these inflammatory lesions (Figure 6d–i), and double staining confirms co-expression of SYK and CD68 in cells present within glomerular crescents (Figure 6j). Occasional SYK+ve CD68–ve cells were also observed within the glomerular tuft, similar to the pattern of staining observed in rat tissue, which may represent other cell types, such as neutrophils, that may be present during the course of AAGN.

## Discussion

The findings reported here are consistent with our previous work showing that SYK inhibition with fostamatinib is effective in both preventing and treating established crescentic glomerulonephritis in anti–glomerular basement membrane disease models.^4^^,^^5^ These data additionally suggest that fostamatinib may inhibit ANCA-induced inflammatory responses, and that it may therefore have therapeutic potential in both pauci-immune and immune-complex–mediated glomerular inflammation. In addition, these new data show that the striking treatment effect of fostamatinib can be achieved with significantly reduced doses compared to our previous work in experimental autoimmune glomerulonephritis, and without significant bone marrow toxicity.

It has been shown that ANCA-induced SYK activation in neutrophils is mediated by signaling through Fc receptors for IgG (FcγR) and the integrin CD18,^8^ and it is likely that disruption of signaling via both these receptors on effector myeloid cells is responsible for the therapeutic effect observed in this study. In particular, both pulmonary and renal disease in this model are characterized by macrophage infiltration, and the unique susceptibility of the WKY rat strain to other forms of experimental glomerulonephritis has been attributed, at least in part, to genetic polymorphisms that control monocyte/macrophage activation, including those in FcγRIII genes.^17^, ^18^, ^19^ In humans, these cells are known to express ANCA autoantigens, including MPO, and they produce proinflammatory mediators following ANCA-stimulation,^20^^,^^21^ which is dependent upon FcγR cross-linking^22^; thus, it is possible that inhibition of SYK-mediated functions in both neutrophils and monocytes is responsible for the reduction in disease severity observed with fostamatinib treatment.

In keeping with a role for SYK activity in monocytes/macrophages in mediating disease, our current and previous immunohistochemical analyses suggest that these cell types are the predominant glomerular leucocyte expressing SYK in both EAV and human AAV, although other cell types, including neutrophils, may be involved. We also observed a significant reduction in macrophage-associated gene transcripts in renal tissue following fostamatinib treatment, including IL-1β, MMP9 and MCP-1. MCP-1 is known to contribute to the pathogenesis of experimental glomerulonephritis and it is present in the glomeruli of patients with AAGN.^23^, ^24^, ^25^ Indeed, urinary levels of this cytokine correlate with disease activity in AAV,^26^ and urinary MCP-1 has been proposed as a novel predictive biomarker in patients,^27^ perhaps supporting potential clinical application of SYK inhibition in AAV.

It is notable that we did not observe a significant effect on circulating MPO-ANCA levels following fostamatinib treatment in this study, in contrast to our previous findings in experimental autoimmune glomerulonephritis, a model of anti—glomerular basement membrane disease, in which we observed inhibition of anti–glomerular basement membrane antibody production.^5^ It is likely that this reflects differences in the time-point at which treatment was initiated in the 2 studies—in experimental autoimmune glomerulonephritis, treatment was started after 18 days, when antibody levels were rapidly increasing, whereas in this study, treatment was introduced after 28 days, when maximal antibody responses were already established, and did not continue beyond the half-life of pre-formed IgG antibody. It is now known that long-term B-cell maturation, survival, and antibody production are highly SYK dependent via both B-cell receptor– and B-cell activating factor (BAFF)–receptor–dependent mechanisms.^28^^,^^29^ B-cell–directed therapy is an established strategy in the treatment of AAV,^30^ and whether disruption of these SYK-dependent B-cell functions with longer-term fostamatinib treatment may provide additional therapeutic benefit in AAV should also be considered.

Off-target drug activity may contribute to some of the therapeutic effects observed in this study. In common with many small molecule kinase inhibitors, R406, the active metabolite of fostamatinib, has been shown to interact with a broad range of targets within the human kinome *in vitro.*^31^^,^^32^ When tested in assays of cellular function, however, R406 demonstrated high selectivity for SYK as determined by phosphorylation of target proteins, despite similar IC_50_ values on isolated kinase assays.^33^ A degree of inhibitory activity against other targets, including Flt3 and JAK2, however is recognized and may have contributed to the effects reported here. The effect of fostamatinib on mature T lymphocytes, which do not usually express SYK, but in which JAK2 signaling contributes to IL-12–induced activation, for example, requires further study. A specific contribution of SYK to neutrophil- and monocyte-mediated glomerular inflammation, however, is suggested by studies in SYK-deficient mice and with alternative pharmacologic inhibitors.^16^^,^^34^^,^^35^

In this study, we have confirmed our previous findings, and those of other groups, that SYK expression is upregulated, and that SYK is activated, at sites of glomerular inflammation in renal AAV.^13^^,^^16^ Our additional *in vivo* data provide further evidence that SYK contributes to disease pathogenesis, and that clinical studies of SYK inhibition should be considered. Given the rapid onset of therapeutic effect observed in the experimental models, SYK inhibition may be a particularly attractive target for gaining rapid control of inflammation in AAV, potentially as a steroid-sparing approach, via its effects on myeloid cell effector functions. Fostamatinib has already shown biological activity and an acceptable toxicity profile in clinical studies in rheumatoid arthritis^36^^,^^37^ and immune thrombocytopenia,^38^ such that the drug was recently approved by the US Food and Drug Administration for treatment of the latter. The preliminary results of a phase II clinical study of SYK inhibition in IgA nephropathy (NCT02112838) were presented in abstract form at the World Congress of Nephrology meeting in April 2019. This study did not show a significant improvement in a primary endpoint of reduction in proteinuria in the whole trial cohort, although predefined subgroup analyses suggested benefit in those patients entering the study with higher levels of proteinuria. This suggests that SYK inhibition may be an effective treatment in more inflammatory forms of glomerulonephritis, such as ANCA-associated glomerulonephritis, and our findings in this preclinical model suggest that this approach should be tested in clinical studies of AAV.

## Methods

A detailed description of all methods—Supplementary Comprehensive Methods—is provided in the Supplementary Material.

### Study approval

All animal procedures were licensed by the Home Office Science Unit, and were conducted in accordance with the UK Animals (Scientific Procedures Act) 1986 and the ARRIVE (Animal Research: Reporting of *In Vivo* Experiments) guidelines. Human tissue samples were provided by the Imperial College NHS Trust Tissue Bank (Application number R15072), in accordance with local research ethics committee approval.

### SYK inhibitors

Fostamatinib and its active metabolite, R406, were provided by Rigel Pharmaceuticals (South San Francisco, CA) and AstraZeneca (London, UK). The details of these molecules have been reported previously.^33^^,^^39^ For *in vivo* studies, animals were treated with fostamatinib 30 mg/kg, 20 mg/kg, or vehicle preparation by twice daily (morning and evening) oral gavage.

### Experimental autoimmune vasculitis

Experimental autoimmune vasculitis was induced by immunizing 6-week-old female Wistar Kyoto rats (n = 8/group) with 1600 mcg/kg human MPO emulsified in CFA supplemented with *Mycobacerium butyricum*. Intraperitoneal pertussis toxin was administered on days 0 and 2, as previously described.^10^ Animals were assessed weekly until 6 weeks after disease induction. At 4 weeks, experimental animals were randomized (with stratification for severity of urinary abnormalities at this time point) to receive vehicle or fostamatinib treatment. Control animals were immunized with CFA alone and followed until 6 weeks (n = 4). To obtain comparative histology at treatment initiation (week 4), an additional group of animals were used after induction of EAV (n = 6).

### Immunohistochemistry and *in situ* hybridization

Immunohistochemistry was performed on formalin-fixed paraffin-embedded tissues using the primary antibodies rat T-SYK N-19 and human T-SYK (both Santa Cruz Biotechnology, Dallas, TX); rat P-SYK Tyr323 (Abcam, Cambridge, UK); rat ED-1 (Bio-Rad, Hercules CA); and human CD68 (Dako Agilent, Santa Clara, CA). ***In situ* hybridization** for SYK mRNA was performed on paraffin-embedded tissues using RNAScope (Advanced Cell Diagnostics, Newark, CA).

### Assessment of renal and lung injury

Lung hemorrhage was graded by visual inspection at the time of cull, and by quantification of hemosiderin-containing and ED-1+ve cells by microscopic assessment using automated image analysis. Hematuria was quantified by dipstick analysis, and proteinuria by the sulphosalicylic acid method.^40^ To assess glomerular injury, 50 consecutive glomeruli in kidney sections were graded as normal or abnormal, and results were expressed as the mean proportion for each animal. Macrophages were immunostained as above, and the number of positive cells per glomerular cross-section were counted in 50 consecutive glomeruli, with results expressed as the mean for each animal. Quantification of histologic changes was performed in a blinded fashion.

### Assessment of autoantibody response and hematological indices

Circulating MPO–ANCA IgG was assayed in serum by enzyme-linked immunosorbent assay. Reactivity to bone marrow–derived leucocytes was confirmed by indirect immunofluorescence on ethanol-fixed cytospin preparations. Deposited antibodies were detected on frozen kidney sections using a fluorescein isothiocyanate–-labeled anti-rat IgG. Hemoglobin concentrations and white blood cell counts were measured in heparinized whole blood samples using an automated analyzer.

### RT-qPCR

RNA extracted from renal cortex was transcribed to cDNA using iScript cDNA synthesis kit (Bio-Rad, Hercules, CA). qPCR was performed using qPCRBIO SyGreen mix (PCR Biosystems, London, UK). Primers used are provided in the Supplementary Methods. qPCR was carried out in duplicate and fold changes were calculated using the 2^-ΔΔCT^ method relative to PGK1.

### Western blot

Lysates of renal cortex or undifferentiated bone marrow cells were resolved by sodium dodecylsulfate–polyacrylamide gel electrophoresis, transferred to nitrocellulose membrane, and probed with the following primary antibodies: glyceraldehyde-3-phosphate dehydrogenase (R&D Systems, Minneapolis, MN), T-SYK (Santa Cruz), and P-SYK (eBioscience, San Diego, CA).

### *In vitro* cell stimulation

Undifferentiated rat bone marrow cells were primed with 4 ng/ml rat TNFα for 30 minutes, then stimulated with either 100 mg/ml MPO-ANCA or control IgG isolated from EAV or healthy rats, respectively. For inhibition experiments, cells were pretreated with R406 and/or vehicle for 30 minutes after TNFα-priming and prior to IgG stimulation. MCP-1 production was measured using a commercially available enzyme-linked immunosorbent assay (BD Biosciences, Oxford, UK), and reactive oxygen species production was measured by CellROX Deep Red assay (Invitrogen, Carlsbad, CA). Results were standardized to allow comparison between each of the biological replicate experiments.

### Statistics

Statistical analysis was conducted using GraphPad Prism 5.0 (GraphPad Software, San Diego, CA). All data are reported as median per group ± interquartile range, unless otherwise stated. Comparison between groups was by Mann-Whitney *U* test and Kruskal-Wallis test with Dunn’s multiple comparison, unless otherwise stated.

## Disclosure

ESM is an employee of and owns stocks/stock options for Rigel Pharmaceuticals. CDP has received a research project grant from GlaxoSmithKline and has a consultancy agreement with Genzyme. FWKT has received research project grants from AstraZeneca Limited, Baxter Biosciences, Boehringer Ingelheim, and MedImmune, and is the principal investigator of an ongoing international clinical trial of an SYK inhibitor in IgA nephropathy (ClinicalTrials.gov NCT02112838), funded by Rigel Pharmaceuticals. He has consultancy agreements with Rigel Pharmaceuticals, Novartis, and Baxter Biosciences. All the other authors declared no competing interests.
