# Supplementary material for: Spleen tyrosine kinase inhibition is an effective treatment for established vasculitis in a pre-clinical model
Source: Kidney Int. 2020 Jun;97(6):1196–207. doi: 10.1016/j.kint.2019.12.014 (PMC7242903; doi:10.1016/j.kint.2019.12.014)
Supplement: Supplementary File (PDF) [file mmc1.pdf]

Spleen tyrosine kinase inhibition is an effective treatment for established vasculitis in a pre-clinical model

Stephen P. McAdoo[1], Maria Prendecki[1], Anisha Tanna[1], Tejal Bhatt[1], Gurjeet Bhangal[1], John McDaid[1], Esteban S. Masuda[2], H. Terence Cook[3], Frederick WK Tam[1]*, Charles D Pusey[1]*

[1]Centre for Inflammatory Disease, Department of Medicine, Imperial College London, London UK.

[2]Rigel Pharmaceuticals, South San Francisco, California USA

*Contributed equally to this work

SUPPLEMENTARY MATERIALS

1. **SUPPLEMENTARY FIGURES**
2. **COMPREHENSIVE METHODS**
3. **ARRIVE CHECKLIST**

SUPPLEMENTARY FIGURES

Figure S1: MPO-ANCA IgG subclasses following fostamatinib treatment in EAV

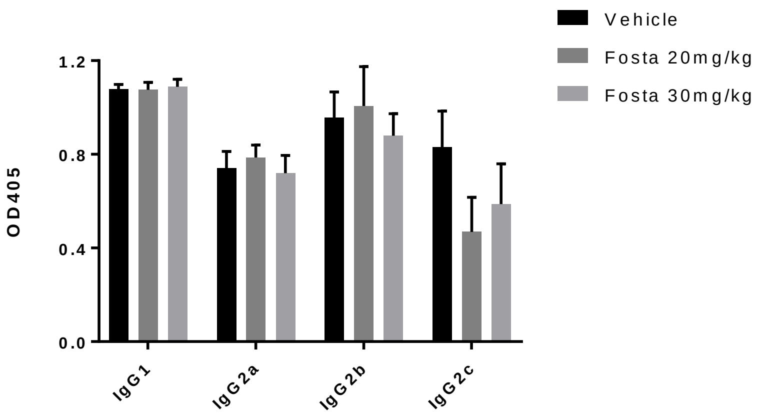

Figure S2: Cell survival following in vitro stimulation assays

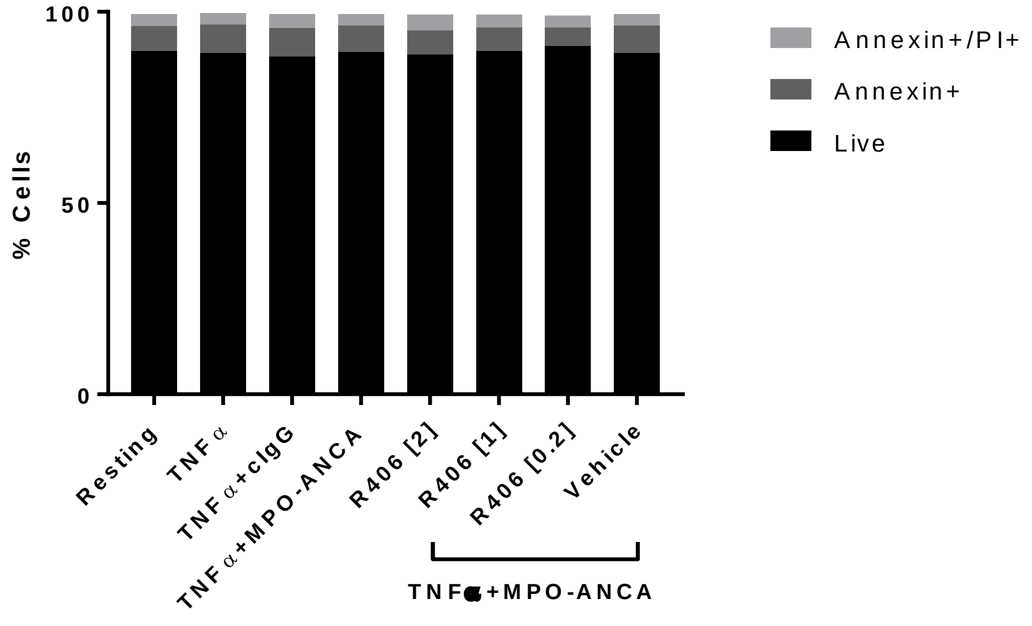

COMPREHENSIVE METHODS

1. SYK inhibitors

R406, the active small molecule inhibitor of SYK, and its oral prodrug, fostamatinib disodium (R788), were provided by Rigel Pharmaceuticals (South San Francisco, California) and AstraZeneca (UK). The details of these molecules have been reported previously[1,2].

Fostamatinib was provided as the disodium salt. For *in vivo* experiments, it was reconstituted in vehicle formulation (0.1% carboxymethylcellulose, 0.1% methylparaben sodium, 0.02% propylparaben sodium, in distilled water, pH 6.5) to a concentration of 8mg/ml, sonicated for 15-20 minutes until dissolved, and then pH re-adjusted to 6.5 with 0.1M HCl. This preparation was stored at 4°C for a maximum of seven days prior to use.

Based on a previous dose-ranging study in nephrotoxic nephritis in Wistar Kyoto (WKY) rats[3], treated animals received 20mg/kg or 30mg/kg body weight, administered by twice daily oral gavage. Control animals received an equivalent volume and schedule of vehicle formulation. Animals were dosed by group (vehicle/20mg/30mg) and in numerical order within group.

For *in vitro* experiments, R406 was reconstituted in 0.02% dimethylsulphoxide (DMSO; Sigma-Aldrich, Poole UK) in cell culture media as detailed below.

2. Animal methods

2.1 Animal study approval

All animal procedures were licensed by the Home Office Science Unit (personal license 70/23027 and project license 70/7104) and conducted in accordance with the UK Animals (Scientific Procedures Act) 1986.

2.2 Animal husbandry

Rats were purchased from Charles River (Margate, UK) and maintained in a pathogen-free animal facility at the Central Biomedical Services Unit, Imperial College London, Hammersmith Hospital campus, in individually-ventilated cages with free access to water and standard laboratory diet. Cage occupancy varied between two to five rats per cage, depending on body weight. Whilst undergoing gavage, animals were provided with wet laboratory diet in case of oesophageal irritation. Animal well-being was assessed daily and monitored weekly by measurements of body weight (Table 1).

Animal	Group	Start Weight (g)	End Weight (g)
1	B	92	179
2	A	144	215
3	C	134	205
4	B	128	192
5	A	117	207
6	C	90	180
7	C	102	202
8	A	89	185
9	B	95	188
10	C	85	165
11	A	90	197
12	B	92	194
13	A	140	208
14	B	134	210
15	A	132	209
16	B	125	208
17	C	140	203
18	C	116	203
19	C	144	202
20	B	103	174
21	A	132	198
22	C	152	208
23	A	137	212
24	B	125	211

Table 1: Summary of animals used (Group A, Vehicle; B, Fosta 20 mg/kg; C, Fosta 30 mg/kg)

2.3 Induction of experimental autoimmune vasculitis

Disease was induced by immunising rats (six week old female WKY rats, approximate weight 120g) with purified human myeloperoxidase (MPO; Calbiochem, Merck Millipore, Darmstadt, Germany) reconstituted in sterile water for injection. A dose of 1600µg/kg was administered, emulsified with an equal volume of complete Freund's adjuvant (CFA; Sigma) with addition of killed *Mycobacterium butyricum*, (to a final concentration of 4mg/ml), with a maximum injection volume of 200µl administered as 2 x 100µl injections to each thigh. Immunised animals also received 500ng of pertussis toxin (Invitrogen, Life Technologies) in PBS intraperitoneally on day zero and day two. For control experiments, animals were immunised with CFA, *Mycobacterium butyricum,* and pertussis toxin using a comparable immunisation schedule.

2.4 Collection of biological specimens from rats

2.4.1 Urine collection

For collection of urine samples, rats were housed overnight, with free access to water and standard laboratory diet, in individual metabolism cages. At the end of the collection, total urine volume was measured, and aliquots were centrifuged at 1500rpm for five minutes to sediment macroscopic debris, and stored at -80°C until use. There was a minimum 48 hour interval between urine collections, in accordance with the terms of the project license.

2.4.2 Serum collection

For collection of serum samples at non-terminal time-points, rats were placed in a warming chamber at 30°C to promote vasodilation, then placed under isofluorane anaesthesia on a warming mat. Whole blood (maximum volume 0.5ml) was collected by superficial tail vessel puncture using a 23 gauge needle. Samples were centrifuged at 1500rpm for five minutes, serum removed and stored at -80°C until use.

2.4.3 Terminal processing of blood and tissues

At the end of the study protocol, animals were placed under isofluorane anaesthesia for final collection of blood and tissues. Blood was obtained either by cardiac puncture using a 10ml pre-heparinised syringe and a 23 gauge needle, or by transection of the great vessels and exsanguination into both plain and heparinised collection tubes.

Tissues were dissected out after the animal had been sacrificed by exsanguination. Lung haemorrhage score was documented by inspection of the lung surfaces before dissection of organs. For each animal, a coronal mid-pole

section of kidney, and a sample of lung, spleen and liver tissue was placed in a histology cassette and transferred to 10% neutral buffered formalin for fixation. A remaining kidney pole was placed in OCT (optimal cutting temperature compound) embedding matrix (ThermoFisher Scientific, Waltham, Massachusetts) on a cork disc, immersed in isopentane and then snap frozen in liquid nitrogen, and stored at -80°C until use. The remaining kidney material, and a sample of lung tissue and spleen tissue were snap frozen in individual cryovials and stored at -80°C until use for PCR or Western blot.

3. Analysis of renal and lung injury

3.1 Haematuria analysis

Haematuria was quantified by dipstick analysis (Multistix 8 SG, Siemens Healthcare Diagnostics, Tarrytown, New York), and expressed as 0 (negative), 0.5 (trace), 1+, 2+ or 3+ for each animal.

3.2 Biochemical analysis of serum and urine

Creatinine concentrations in serum samples were kindly measured by Dr Olatunji Rowland and Dr John Morris in the Department of Clinical Biochemistry, Hammersmith Hospital UK, using an AU700 analyser (Olympus, Southend, UK).

Proteinuria was quantified using sulphosalicylic acid method. Urine samples, diluted between 1:3 and 1:100 in water, were added to a 96-well microtitre plate in triplicate, to which 10µl of 25% sulphosalicylic acid was added to two of three replicates, and 10µl of water to the third replicate (providing a 'blank' reading for that sample). Known quantities of bovine serum albumin (BSA; Sigma) in water were used to define a standard curve, and absorbance read at 450nm on microplate reader and dedicated software (Biotek EL800; Gen5 Analysis Software; both Biotek Instruments Ltd, Potton, UK). The protein concentration in each sample was calculated from a regression equation described by the standard curve, and the 24 hour protein excretion rate was calculated by multiplying by the total urine volume.

3.3 Haematological analysis

Haemoglobin concentrations and white blood cell and platelet counts were measured in heparinised whole blood samples using an automated analyser (XE-2100, Sysmex, Milton Keynes, UK) in the Department of Clinical Haematology, Hammersmith Hospital UK.

3.4 Renal histology

Kidney tissue collected at the time of sacrifice was fixed in 10% neutral buffered formalin overnight, then transferred to 70% ethanol and processed to paraffin blocks. 4µm sections were cut on a rotary microtome and stained with haematoxylin and eosin (H&E) and periodic acid Schiff (PAS) for assessment of renal injury. For quantification of renal injury in EAV, 50 consecutive glomeruli were assessed by light microscopy in a blinded fashion, and graded as normal or abnormal. Results are expressed as the percentage of abnormal glomeruli per animal.

3.5 Immunohistochemistry for leucocyte markers

Immunostaining for ED1 positive cells was performed on formalin-fixed paraffin embedded kidney and lung sections. For details of immunostaining methods, see section 6. ED1 positive cell infiltrate in glomeruli was quantified in a blinded fashion by counting the number of ED-1 positive cells in 50 consecutive glomeruli in each section, and expressed as the mean number of cells per glomerular cross section (GCS) for each animal. ED1 positive cell infiltration in the lung was quantified in a blinded fashion by counting the mean number of ED-1 positive cells in five high power field (HPF) examinations of each section.

3.6 Macroscopic assessment of lung injury

A semi-quantitative scoring system was used to grade the severity of lung haemorrhage by visual inspection at the time of cull. Lungs were graded as follows: 0 points – normal macroscopic lung appearances; one point if fewer than ten petechiae were visible; two points if ten to 20 petechiae were visible; three points if more than 20 petechiae were visible; four points if large areas of infarction or haemorrhage were evident. Representative lung appearances are shown in Figure 1.

Figure 1: Scoring system to quantify lung haemorrhage (LH) severity.

The lungs surfaces were inspected at the time of sacrifice, and scored in accordance with the number of visible petechiae on the lung surface: zero points – normal macroscopic lung appearances (Panel A); one point if fewer than ten visible petechiae (Panel B); two points if ten to 20 visible petechiae (Panel C); three points if more than 20 visible petechiae (not shown); four points if large areas of infarction or haemorrhage were evident (Panel D; PH, pulmonary haemorrhage).

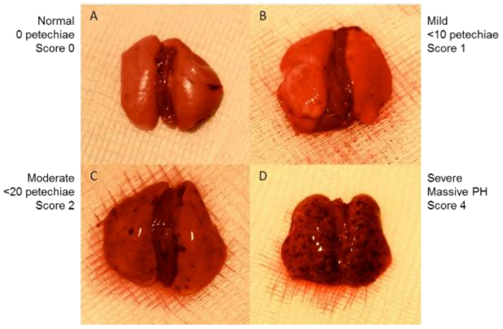

3.7 Microscopic scoring of lung injury

Lung tissue was collected for histological analysis and processed as per kidney tissue (section 3.4). 4µm paraffin-embedded sections were stained with Perls' Prussian blue without counterstain, to enable identification of haemosiderin-laden cells. These were quantified by a blinded observer using automated image analysis software (ImagePro Plus, Media Cybernetics, Rockville, Maryland) to measure the proportion of Perls' stained cells across five random high-power fields of lung sections from each animal, and expressed as the mean proportion per high power field per animal.

4. Assessment of humoral responses

4.1 ELISA for anti-MPO antibodies

Anti-MPO antibody levels in rat sera were measured using direct ELISA[4,5]. Wells were coated with purified human MPO (Calbiochem) at a concentration of 1.33µg/ml in carbonate buffer and stored at 4°C overnight. The following day, plates were washed three times in 0.1% PBS/T, and non-specific binding sites blocked by incubating the wells with 1% BSA in 0.1% PBS/T for one hour. After three washes in 0.1% PBS/T, 100µl of serum samples (diluted 1:100 to 1:100000 in PBS/T plus 1% BSA) were applied in duplicate, with a negative control 'blank' that did not contain serum. Plates were incubated at 37°C for one hour and then washed three times in 0.1% PBS/T. 100µl of rabbit anti-rat IgG with ALP conjugate in 0.1% PBS/T plus 1% BSA (dilution 1:1000) was then added for one hour at 37°C. After a wash cycle in 0.1% PBS/T, 100µl of p-nitrophenyl phosphate was applied to each well. The colour change was monitored, and then quantified on a microplate reader at 405nm. A co-efficient of variance of 10% between sample replicates was accepted. Historical pooled sera were used to develop a standard curve from which subsequent samples could be assigned an interpolated value in arbitrary units.For quantification of IgG subclasses, a modification of this protocol was used, substituting secondary antibodies specific for rat IgG1, IgG2a, IgG2b, and IgG2c (BioLegend, San Diego CA).

4.2 Direct immunofluorescence for deposited antibodies

IgG deposition in glomeruli was assessed by direct immunofluorescence using snap frozen renal tissue obtained at the time of cull. Frozen kidney sections were cut on a cryostat at 5µm thickness and placed on poly-L-Lysine coated slides (Leica Biosystems, Milton Keynes, UK). After fixation in acetone for ten minutes, the slides were air dried. If not used immediately, the slides were stored at -80°C in sealed boxes containing silica crystals. The slides were blocked with 20% normal rabbit serum (Dako Agilent, Santa Clara CA) for 30 minutes at room temperature. After washing in PBS, the slides were incubated with FITC (fluorescein isothiocyanate)-conjugated rabbit anti-rat IgG (Sigma) at 1:100 dilution in PBS for one hour at room temperature in a humidified chamber. Following two ten minute washes in PBS, the sections were mounted in PBS/Glycerol (Citifluor, London, UK). For quantification, 20 consecutive glomeruli on each section were inspected by fluorescence microscopy in a blinded fashion, and intensity graded 0/1+/2+/3 in arbitrary units, and expressed as the mean intensity per glomerulus

for each animal. Positive control tissue taken from animals after induction of nephrotoxic nephritis[6] were used for this analysis.

5. *In vitro* cell stimulation studies

5.1 Derivation of undifferentiated bone marrow cells

After sacrifice as described in section 2.4.3, healthy WKY rat femurs were isolated and transferred to a tissue culture hood in ice-cold sterile HBSS solution. The bones were cleaned of hair and soft tissue, washed twice in 70% ethanol, and rinsed again in HBSS. Both ends of the cleaned bones were then cut, and bone marrow cells flushed out with a 20 gauge needle using 10ml of cold HBSS per bone, and collected into a 50ml centrifuge tube. Cells were then centrifuged at 1500rpm for five minutes at 4°C, and re-suspended in 10ml HBSS buffer. The 50ml centrifuge tube was then placed in a CO_2 incubator in a horizontal position for 10min to hypotonically lyse red blood cells. The cells were then centrifuged at 1500rpm for five minutes at 4°C, the supernatant aspirated, and the cell pellet re-suspended in serum-free cell culture medium (RPMI supplemented with 2% penicillin and streptomycin, and 2mM L-glutamine).

5.2 Derivation of MPO-ANCA and control rat IgG

Rat serum collected from historical animals six weeks after induction of EAV was diluted in a 1:1 ratio in binding buffer (20mM sodium phosphate, pH 7.0) and sterile filtered (0.2 nm filter), then passed through a 'HiTrap' protein G sepharose column (GE), and eluted by 0.1M glycine HCl (pH 2.7) into Tris HCl (pH 9). Total IgG concentrations were quantified by spectrophotometry (absorbance 260nm) and aliquots with highest concentration were pooled, dialysed overnight in a 10,000 MW cut-off cartridge against sterile PBS, and total IgG concentrations then re-quantified as above.

5.3 Stimulation of bone marrow cells

Undifferentiated bone marrow cells were suspended in serum-free cell culture medium to a final concentration of 20 million cells per ml in 24 well cell culture plate, and primed by 30 minutes exposure to rat TNFα (final concentration 4ng/ml; Sigma), and then stimulated with either MPO-ANCA IgG (100µg/ml) or control IgG (100µg/ml). For SYK inhibitor studies, cells were pre-incubated with vehicle (0.02% DMSO) or 0.2µM, 1µM and 2µM R406 in vehicle for 30 minutes (after TNFα-priming and prior to IgG stimulation). Cell culture supernatants were collected after four hours and stored at -80°C until used. Experiments were conducted in technical (cell culture) triplicate and with four biological replicates.

5.4 Annexin-PI assay

Annexin PI (Biolegend) viability assay was carried out on cells following stimulation as detailed. Cells were recovered, washed twice in cold cell staining buffer and resuspended in binding buffer at $1x10^7$ cells/ml. 5µl of Annexin V and 10 µl of PI solutions were added to 100 µl of cell suspension and incubated at room temperature for 15 minutes followed by addition of excess binding buffer and analysis by flow cytometry.

5.5 Measurement of monocyte chemoattractant protein 1 (MCP-1)

MCP-1 concentrations in cell culture supernatants were measured using a commercially available sandwich ELISA development kit (OptEIA Rat MCP-1 ELISA Set, BD Biosciences) used according to the manufacturer's instructions. A 96-well ELISA plate was coated with 100µl of capture antibody at a dilution of 1:250 in carbonate buffer, sealed and stored overnight at 4°C (or for a maximum of four days) until use. For use, the capture antibody solution was removed, wells washed thrice in 0.1% PBS/T, and 200µl of assay diluent (10% FCS in PBS) was added to each well for one hour at room temperature to block non-specific binding sites on the surface of the plate. After three washes in 0.1% PBS/T, 100µl of samples (neat or diluted up to 1:5000 in assay diluent, as necessary), known standards and negative control samples (containing assay diluent alone) were added to wells in duplicate and incubated for one hour at room temperature. After three further washes in 0.1% PBS/T, 100µl of assay diluent containing biotinylated detection antibody (1:500) and streptavidin-HRP conjugate (1:250) was added to each well for one hour, and wells washed five times. A solution of 3,3',5,5'-tetramethylbenzidine (TMB; Cambridge Bioscience, Cambridge UK) and hydrogen peroxide was prepared, and 100µl added to each well. The plate was incubated in the dark for approximately ten minutes before stopping the reaction by adding 50µl of 2M sulphuric acid. The absorbance was measured at 450nm on a microplate reader. Adjusted absorbance was calculated by subtracting the background absorbance of the 'blank' sample. A best-fit standard curve was generated from the absorbances of the recombinant MCP-1 reference series using four variable non-parametric regression modelling. Concentrations of MCP-1 in samples could then be calculated from the raw blank-subtracted absorbances using the resultant regression equation. A co-efficient of variance of <10% between sample replicates was accepted. As absolute amounts of MCP-1 production were variable between each biological replicate experiment, results were standardised and expressed as a percentage maximum MCP-1 production for a given experiment.

5.6 Reactive Oxygen Species (ROS) assay

For detection of ROS the CellRox™ Deep Red flow cytometry kit (Molecular Probes, Life Technologies) was used according to the manufacturer's protocol. Cells were stimulated as detailed, followed by addition of CellROX to a working concentration of 5 µM, and incubation for 30 minutes at 37°C. Cells were washed, fixed in 1% paraformaldehyde and analysed by flow cytometry. For a positive control cells were incubated with 200 µM Tert-butyl hydroperoxide.

6. Immunohistochemistry (IHC)

6.1 Pre-treatment of paraffin sections

All IHC in this study was performed on formalin-fixed paraffin embedded tissue sections. Paraffin wax embedding and sectioning of formalin-fixed animal tissues was performed by Ms Lorraine Lawrence (Department of Leukocyte Biology, Imperial College London). Sections were cut on a rotary microtome to a thickness of 4µm.

Sections were placed in two sequential baths of xylene, to remove wax, before passage through graded ethanol concentrations and finally water. De-waxed sections underwent heat-induced epitope retrieval in 0.1M sodium citrate buffer, pH 6.0. The sections were then sequentially blocked for endogenous peroxidase activity by submerging in 0.3% hydrogen peroxide from ten minutes, and rinsed in PBS. A 20% serum solution was applied for 30 minutes (See Table 1 for details) to block non-specific binding of subsequent antibodies.

6.2 Primary antibodies

The blocking antibody was tapped off, and primary antibodies added at the appropriate dilution in sufficient volume to cover the tissue section, typically 50-200µl. Slides were placed in a covered, humidified staining chamber to minimise evaporation and left to incubate for the appropriate duration (Table 1). For SYK IHC, negative controls comprised omission of primary antibody and, where available, use of primary antibody pre-incubated with the cognate immunising peptide at a 1:1 ratio. Omission of primary antibody was used for IHC protocols that were already established in our laboratory (e.g. ED-1).

6.3 Detection

Three methods were used for detection of primary antibodies in tissue sections:

(i) A commercial secondary antibody detection system (EnVision, Dako) was used for the detection of the total and phosphorylated SYK antibodies (Table 1). Primary antibody was tapped off and slides washed two times for five minutes in PBS. Sufficient EnVision reagent was then added to completely cover the tissue section and the slide placed in the humidified incubation chamber for 30 minutes. The reagent was then tapped off and a further two PBS washes performed.

(ii) For rat ED1 staining, excessive non-specific background staining within glomeruli was observed using the EnVision system. In these instances, a biotinylated rabbit anti-mouse secondary antibody (as detailed in Table 1) was incubated with the sections for one hour. The sections were then washed twice in PBS, and incubated with an Extravadin-HRP conjugate (Sigma) at a dilution of 1:100 for 30 minutes, then washed thrice in PBS.

A 3,3’-diaminobenzidine (DAB) chromagen solution was prepared by mixing DAB+ chromagen with DAB+ substrate buffer in a ratio of 1:50 (Dako). This was applied to tissue sections for 30 seconds to five minutes depending on the primary antibody. The reaction was terminated by placing slides in PBS. Slides were then rinsed in water and counterstained by immersion for 30 seconds in filtered Harris haematoxylin (CellPath, Powys, UK). After rinsing, excess stain was removed by brief (<1 second) immersion in acid-alcohol solution (1% HCl in 70% ethanol). Sections were then washed in water and dehydrated by sequential passage through ascending alcohol concentrations to xylene. Slides were then mounted with DPX (Distrene, Plasticiser, Xylene; VWR International, Lutterworth, UK) and glass coverslips and allowed to dry before examination.

6.4 Double staining

For double staining for SYK and CD68/ED-1, in both rat and human tissue, sections were first stained for T-SYK as described above. Following development in DAB, the sections were sequentially rinsed, re-subjected to heat-induced antigen retrieval (0.1M citrate buffer, pH 6.0), blocked with 20% rabbit or goat serum (Dako), incubated with mouse anti-rat ED-1 primary antibody (Serotec) or mouse anti-human CD68 (Dako) at room temperature for 1 hour, washed, then incubated with a biotinylated rabbit or goat anti-mouse immunoglobulin secondary antibody (Sigma; dilution 1:100) for one hour, then ALP-conjugated streptavidin (Roche Diagnostics; dilution 1:100) for 30 minutes, before final development using the Vector Blue ALP Substrate Kit III (Vector Labs, Peterborough, UK) according to manufacturer’s specifications. Slides were mounted in AquaPerm (ThermoFisher Scientific) without haematoxylin counterstaining, and coverslips placed.

**TARGET PROTEIN (SPECIES)**	**BLOCKING SOLUTION**	**PRIMARY ANTIBODY**	**PRIMARY ANTIBODY DILUTION AND INCUBATION**	**SECONDARY ANTIBODY (FOR SINGLE STAINING)**
Total SYK (Rat)	20% Goat Serum (Dako)	SYK-N19 Santa-Cruz #1077 Rabbit polyclonal	1:1000 1h, room temperature	Dako-EnVision HRP-linked polymer anti-rabbit
Phosphorylated SYK (Rat)	20% Goat Serum (Dako)	P-SYK Tyr323 Abcam #63515 Rabbit polyclonal	1:50 2h, room temperature or overnight,	Dako-EnVision HRP-linked polymer anti-rabbit

ED1<br><br>(Rat)	20% Rabbit Serum (Dako)	Clone ED1<br><br>Serotec<br><br>Mouse monoclonal	1:500<br><br>1h, room temperature	Biotinylated rabbit anti-mouse immunoglobulin (Sigma; dilution 1:100);<br><br>Followed by Extravadin-HRP (Sigma; dilution 1:100)
Total SYK<br><br>(Human)	20% Goat Serum (Dako)	Clone 4d10<br><br>Santa-Cruz<br><br>Mouse monoclonal	1:250<br><br>Overnight, 4°C	Dako-EnVision HRP linked polymer anti- mouse
CD68<br><br>(Human)	20% Goat Serum (Dako)	Clone PGM1<br><br>Dako<br><br>Mouse monoclonal	1:50<br><br>1h, room temperature	Dako-EnVision HRP linked polymer anti- mouse

Table 2: Summary of antibodies used for immunohistochemistry.

7. RNAScope™

RNAscope™ *in situ* hybridization was carried out on FFPE tissue sections according to the manufacturer's instructions (Advanced Cell Diagnostics, Newark CA). Freshly cut sections were baked for 1 hour at 60°C then de-waxed as described for IHC. Antigen retrieval was for 15 minutes in boiling TR reagent. Sections were then incubated with hydrogen peroxide for 10 minutes at room temperature followed by Protease Plus at 40°C in a HybEZ™ oven. Sections were then sequentially incubated with hybridization probed for SYK, PPIB (positive control) or DapB (negative control), followed by six amplification probes; these steps were performed at 40°C in a HybEZ™ oven. Signal was detected using 2.5 HD red detection reagents. Sections were washed in distilled water to stop the reaction and counterstained using Mayer's haematoxylin. Sections were dried for one hour at 60°C, briefly dipped in xylene and mounted using aqueous mounting media.

8. Polymerase chain reaction

Approximately 50mg of rat renal cortex (from animals with EAV, animals with EAV treated with fostamatinib or vehicle, andcontrol rats immunised with CFA alone (n=4/group minimum)) was homogenised in 1ml of TRIzol reagent (Invitrogen, Carlsbad CA) using a dounce homogeniser and centrifuged at 12000xg to remove particulate matter. Total RNA was purified using the Direct-zol RNA Miniprep protocol (Zymo, Irvine CA). RNA yield and quality was quantified using Nanodrop and 1 mcg of RNA transcribed to cDNA using iscript cDNA

synthesis kit (Bio-Rad, Hercules CA). qPCR was performed using qPCRBIO SyGreen mix (PCR Biosystems, London UK) according to the manufacturer's instructions on an Eppendorf realplex mastercycler. Primers used are given in Table 3. T_m was 61°C and primer concentration 400 nm. qPCR was carried out in duplicate and fold changes calculated using the $2^{-\Delta\Delta CT}$ method relative to GAPDH. [Additiion Primers].

Target	Forward	Reverse
MCP-1	TCCACCACTATGCAGGTCTC	GGGCATTAACTGCATCTGGCT
MMP9	TCCAGCATCTGTATGGTCGTG	GCAGTGGGACACATAGTGGG
CCL3	TGCTGCTTCTCCTATGGACG	TTGGTCAGGAAAATGACACCCG
TNFα	ATGGGCTCCCTCTCATCAGT	GCTATGGCAACTGTCCCTGA
IL-6	AGAGACTTCCAGCCAGTTGC	AGTCTCCTCTCCGGACTTGT
IL-2	CCAAGCAGGCCACAGAATT	TCCAGCGTCTTCCAAGTGAA
IL-1β	CCTTGTGCAAGTGTCTGAAGC	CAGGTCATTCTCCTCACTGTCG
IL-4	GTACCAGACGTCCTCACGGC	TCAGACCGCTGACACCTCTA
SYK	AGTTCAAATGCCCATCCTGT	GTGGGGGAGGCTTTTTGT
PGK1[7]	ATGCAAAGACTGGCCAAGCTAC	AGCCACAGCCTCAGCATATTTC

Table 3: Primers used for PCR

9. Western Blot

Tissue lysates were prepared by homogenizing an approximately 5 mg piece of renal cortex in 300 µl of ice cold NP40 lysis buffer (Invitrogen) supplemented with protease inhibitors, PMSF (1 mM) and sodium orthovanadate (1 mM). For cell lysates, $1x10^6$ cells were lysed for 5 minutes in 300 µl of ice cold NP40 lysis buffer supplemented as previously. Samples were centrifuged for 10 minutes at 12,000g 4°C and supernatant transferred to fresh microcentrifuge tubes. Protein concentration was quantified using BCA assay (Pierce). Samples were denatured by boiling for 3 minutes in Laemelli buffer (4% SDS, 10% 2-mercaptoethanol, 20% glycerol, 0.004% bromophenol blue, 0.125M Tris HCl, pH 6.8) and resolved on Bis-Tris 4-12% NuPage gels (Invitrogen). Protein was transferred to PVDF membrane (Amersham), blocked for one hour in 5% BSA in TBS/0.1% Tween 20 and incubated overnight in P-SYK antibody (1 µg/ml, P-SYK348, eBioscience, moch1cht). Membranes were washed then incubated with anti-mouse IgG HRP-conjugated secondary antibody (1:5000, BioRad) for one hour followed by further washes and signal detected using ECL detection reagents (Thermofisher) and photographic film (Amersham). Membranes were then stripped of antibody by incubating with stripping buffer (15% glycine, 1% SDS, 0.1% Tween-20, pH 2.2) and re-probed for T-SYK (Santa-Cruz) and GAPDH (R+D) as above but with 5% marvel milk solution in place of BSA and secondary antibodies of the appropriate species.

10. Statistics

Graphs were constructed and statistical analysis conducted using Prism 5.0 (GraphPad Software Inc., San Diego, California). Unless otherwise stated, all data are reported as median with interquartile range. Whenever a statistical test was used, this is indicated in the text. The means of two or more normally distributed variables were compared with a t-test or analysis of variance (ANOVA) respectively. For non-parametric datasets, or where ≤8 samples were available, Mann-Whitney U, Kruskal-Wallis or Friedman tests were used to assess the difference between 2, >2 and >2 (repeated measures) groups respectively, with Dunn's post-hoc test to compare individual groups. All tests were two-tailed.

REFERENCES

1. Braselmann S, Taylor V, Zhao H, et al. R406, an orally available spleen tyrosine kinase inhibitor blocks fc receptor signaling and reduces immune complex-mediated inflammation. *The Journal of pharmacology and experimental therapeutics.* 2006;319(3):998-1008.

2. Bahjat FR, Pine PR, Reitsma A, et al. An orally bioavailable spleen tyrosine kinase inhibitor delays disease progression and prolongs survival in murine lupus. *Arthritis and rheumatism.* 2008;58(5):1433-1444.

3. Smith J, McDaid JP, Bhangal G, et al. A spleen tyrosine kinase inhibitor reduces the severity of established glomerulonephritis. *Journal of the American Society of Nephrology : JASN.* 2010;21(2):231-236.

4. Little MA, Smyth CL, Yadav R, et al. Antineutrophil cytoplasm antibodies directed against myeloperoxidase augment leukocyte-microvascular interactions in vivo. *Blood.* 2005;106(6):2050-2058.

5. Little MA, Smyth L, Salama AD, et al. Experimental autoimmune vasculitis: an animal model of anti-neutrophil cytoplasmic autoantibody-associated systemic vasculitis. *The American journal of pathology.* 2009;174(4):1212-1220.

6. Tam FW, Smith J, Morel D, et al. Development of scarring and renal failure in a rat model of crescentic glomerulonephritis. *Nephrology, dialysis, transplantation : official publication of the European Dialysis and Transplant Association - European Renal Association.* 1999;14(7):1658-1666.

7. Langnaese K, John R, Schweizer H, Ebmeyer U, Keilhoff G. Selection of reference genes for quantitative real-time PCR in a rat asphyxial cardiac arrest model. *BMC Mol Biol.* 2008;9:53.

The ARRIVE Guidelines Checklist

Animal Research: Reporting In Vivo Experiments

Carol Kilkenny[1], William J Browne[2], Innes C Cuthill[3], Michael Emerson[4] and Douglas G Altman[5]

[1]The National Centre for the Replacement, Refinement and Reduction of Animals in Research, London, UK, [2]School of Veterinary Science, University of Bristol, Bristol, UK, [3]School of Biological Sciences, University of Bristol, Bristol, UK, [4]National Heart and Lung Institute, Imperial College London, UK, [5]Centre for Statistics in Medicine, University of Oxford, Oxford, UK.

	ITEM	RECOMMENDATION	Section/ Paragraph
Title	1	Provide as accurate and concise a description of the content of the article as possible.	Title
Abstract	2	Provide an accurate summary of the background, research objectives, including details of the species or strain of animal used, key methods, principal findings and conclusions of the study.	Abstract
**INTRODUCTION**			
Background	3	a. Include sufficient scientific background (including relevant references to previous work) to understand the motivation and context for the study, and explain the experimental approach and rationale.<br>b. Explain how and why the animal species and model being used can address the scientific objectives and, where appropriate, the study's relevance to human biology.	Throughout introduction and discussion
Objectives	4	Clearly describe the primary and any secondary objectives of the study, or specific hypotheses being tested.	Introduction Para 2
**METHODS**			
Ethical statement	5	Indicate the nature of the ethical review permissions, relevant licences (e.g. Animal [Scientific Procedures] Act 1986), and national or institutional guidelines for the care and use of animals, that cover the research.	Concise Methods Para 1<br>Extended methods Section 2.1

Study design	6	For each experiment, give brief details of the study design including:<br>a. The number of experimental and control groups.<br>b. Any steps taken to minimise the effects of subjective bias when allocating animals to treatment (e.g. randomisation procedure) and when assessing results (e.g. if done, describe who was blinded and when).<br>c. The experimental unit (e.g. a single animal, group or cage of animals).<br>A time-line diagram or flow chart can be useful to illustrate how complex study designs were carried out.	a: Concise methods Para 3<br>b: Concise methods Para 3, Para 5, Extended Methods Section 3<br>c: Single animal, as presented throughout results
Experimental procedures	7	For each experiment and each experimental group, including controls, provide precise details of all procedures carried out. For example:<br>a. How (e.g. drug formulation and dose, site and route of administration, anaesthesia and analgesia used [including monitoring], surgical procedure, method of euthanasia). Provide details of any specialist equipment used, including supplier(s).<br>b. When (e.g. time of day).<br>c. Where (e.g. home cage, laboratory, water maze).<br>d. Why (e.g. rationale for choice of specific anaesthetic, route of administration, drug dose used).	a: Concise methods Para 3, Extended Methods Sections 1, 2.3, 2.4<br>b: Concise methods Para 3, Extended Methods Section 1<br>c: Extended Methods Section 2.2, 2.4<br>d: Extended Methods Section 1, Discussion Para 1
Experimental animals	8	a. Provide details of the animals used, including species, strain, sex, developmental stage (e.g. mean or median age plus age range) and weight (e.g. mean or median weight plus weight range).<br>b. Provide further relevant information such as the source of animals, international strain nomenclature, genetic modification status (e.g. knock-out or transgenic), genotype, health/immune status, drug or test naïve, previous procedures, etc.	a: Concise methods Para 3, Extended Methods Section 2.3<br>b: Extended methods 2.2

The ARRIVE guidelines. Originally published in *PLoS Biology*, June 2010[1]

Housing and husbandry	9	Provide details of:<br>a. Housing (type of facility e.g. specific pathogen free [SPF]; type of cage or housing; bedding material; number of cage companions; tank shape and material etc. for fish).<br>b. Husbandry conditions (e.g. breeding programme, light/dark cycle, temperature, quality of water etc for fish, type of food, access to food and water, environmental enrichment).<br>c. Welfare-related assessments and interventions that were carried out prior to, during, or after the experiment.	a: Extended methods 2.2<br>b: Extended methods 2.2<br>c: Extended methods 2.2
Sample size	10	a. Specify the total number of animals used in each experiment, and the number of animals in each experimental group.<br>b. Explain how the number of animals was arrived at. Provide details of any sample size calculation used.<br>c. Indicate the number of independent replications of each experiment, if relevant.	a: Concise methods Para 3<br>b: Group size determined by previous experience in our laboratory; *a priori* sample size calculations were not performed<br>c: Not applicable
Allocating animals to experimental groups	11	a. Give full details of how animals were allocated to experimental groups, including randomisation or matching if done.<br>b. Describe the order in which the animals in the different experimental groups were treated and assessed.	a: Concise methods Para 3<br>b: Extended Methods Section 1
Experimental outcomes	12	Clearly define the primary and secondary experimental outcomes assessed (e.g. cell death, molecular markers, behavioural changes).	Throughout Results
Statistical methods	13	a. Provide details of the statistical methods used for each analysis.<br>b. Specify the unit of analysis for each dataset (e.g. single animal, group of animals, single neuron).<br>c. Describe any methods used to assess whether the data met the assumptions of the statistical approach.	a: Concise Methods Para 10, Extended Methods Section 10<br>b: Throughout Results; Extended Methods Section 3<br>c: Non-parametric tests appropriate given small group size (n=8)
RESULTS			
Baseline data	14	For each experimental group, report relevant characteristics and health status of animals (e.g. weight, microbiological status, and drug or test naïve) prior to treatment or testing. (This information can often be tabulated).	Extended Methods Section 2.2
Numbers analysed	15	a. Report the number of animals in each group included in each analysis. Report absolute numbers (e.g. 10/20, not 50%[2]).<br>b. If any animals or data were not included in the analysis, explain why.	a: Throughout Results<br>b: Not applicable; all animals reported

Outcomes and estimation	16	Report the results for each analysis carried out, with a measure of precision (e.g. standard error or confidence interval).	a: Throughout Results
Adverse events	17	a. Give details of all important adverse events in each experimental group.<br>b. Describe any modifications to the experimental protocols made to reduce adverse events.	a: Not applicable; no important adverse events observed<br>b: Extended Methods Section 2.2 (pre-emptive dietary modification)
DISCUSSION			
Interpretation/ scientific implications	18	a. Interpret the results, taking into account the study objectives and hypotheses, current theory and other relevant studies in the literature.<br>b. Comment on the study limitations including any potential sources of bias, any limitations of the animal model, and the imprecision associated with the results[2].<br>c. Describe any implications of your experimental methods or findings for the replacement, refinement or reduction (the 3Rs) of the use of animals in research.	a: Throughout Discussion<br>b: Throughout Discussion<br>c: Not applicable
Generalisability/ translation	19	Comment on whether, and how, the findings of this study are likely to translate to other species or systems, including any relevance to human biology.	Throughout Discussion
Funding	20	List all funding sources (including grant number) and the role of the funder(s) in the study.	Funding Acknowledgements

References:
1. Kilkenny C, Browne WJ, Cuthill IC, Emerson M, Altman DG (2010) Improving Bioscience Research Reporting: The ARRIVE Guidelines for Reporting Animal Research. *PLoS Biol* 8(6): e1000412. doi:10.1371/journal.pbio.1000412
2. Schulz KF, Altman DG, Moher D, the CONSORT Group (2010) CONSORT 2010 Statement: updated guidelines for reporting parallel group randomised trials. *BMJ* 340:c332.
